# Draft Genome Sequence of Cellulolytic and Xylanolytic *Cellulomonas* sp. Strain B6 Isolated from Subtropical Forest Soil

**DOI:** 10.1128/genomeA.00891-16

**Published:** 2016-08-25

**Authors:** Florencia Piccinni, Yanina Murua, Silvina Ghio, Paola Talia, Máximo Rivarola, Eleonora Campos

**Affiliations:** a Instituto de Biotecnología, CICVyA, INTA, Buenos Aires, Argentina; b Instituto de Suelos, CIRN, INTA, Buenos Aires, Argentina; c Facultad de Ingeniería y Ciencias, Exactas, UADE, Buenos Aires, Argentina; d Consejo Nacional de Investigaciones Científicas y Técnicas (CONICET), Buenos Aires, Argentina

## Abstract

*Cellulomonas* sp. strain B6 was isolated from a subtropical forest soil sample and presented (hemi)cellulose-degrading activity. We report here its draft genome sequence, with an estimated genome size of 4 Mb, a G+C content of 75.1%, and 3,443 predicted protein-coding sequences, 92 of which are glycosyl hydrolases involved in polysaccharide degradation.

## GENOME ANNOUNCEMENT

Cellulases and xylanases are widely used in textile, animal feed, food, and paper industries. They also play a key role in the production of cellulosic ethanol (1).

*Cellulomonas* sp. strain B6 (available from Argentine collection of microorganisms as IMIZA:CEB6) was isolated from the first 10-cm layer of a preserved native subtropical forest soil sample (26°01′34′′S 54°26′59′′W) (2). It is a Gram-positive, rod-shaped, aerobic isolate that can grow on lignocellulosic biomass, such as sugarcane residue, as a sole carbon source. Its secreted protein extract presented cellulose- and xylan-degrading activities (our unpublished data). Based on 16S rRNA analysis, strain B6 formed a cluster with *Cellulomonas flavigena* (accession no. AF140036.1) and *Cellulomonas persica* (accession no. NR_024913.1). The genomes of *Cellulomonas flavigena* DSM 20109 and B6 show an average nucleotide Identity (ANI) value of 81.99%, suggesting that they are different species.

Genomic DNA of *Cellulomonas* sp. strain B6 was extracted from a 24-h culture in LB broth by a commercial extraction kit (Wizard genomic DNA extraction kit; Promega) and sequenced using the Illumina MiSeq platform. The data comprised 1,532,556 paired-end reads of 500 bp, resulting in 83-fold genome coverage. The raw reads were subjected to trimming using Trimmomatic version 0.33 (3) and assembled *de novo* using Celera Assembler version 8.2 (4), followed by the SPAdes genome assembler version 3.5.0 (5), generating 279 contigs, with a total length of 4,042,435 bp (*N*_50_, 24,612 bp) and a G+C content of 75.1%, consistent with the genus.

Gene prediction and functional analysis were carried out using the Rapid Annotations using Subsystems Technology (RAST) server version 2.0 (6) and the NCBI Prokaryotic Genome Annotation Pipeline (http://www.ncbi.nlm.nih.gov/genome/annotation_prok/). Using the NCBI pipeline, 3,691 genes, including 3,443 protein-coding sequences, 50 tRNA, and a set of full-length 5S, 23S, and 16S rRNA gene sequences, were predicted. A noncoding RNA (ncRNA) of an RNase P (ATM99_11600) was also predicted. Similar results were obtained by RAST. A comparison of a representative set of *FigFam* protein-coding genes from *Cellulomonas* sp. B6 to other bacterial sequences available in RAST identified *Cellulomonas flavigena* DSM 20109 (score, 413) and *Sanguibacter keddieii* DSM 10542 as the closest neighbors.

Utilizing all functional annotations from CAZy (http://www.cazy.org/) (7) and dbCAN (http://csbl.bmb.uga.edu/dbCAN/) (8), 92 sequences encoding potential glycosyl hydrolases (GH) were identified, including six endo-β-1,4-glucanases (two GH5 and four GH9), two exo-glucanases (GH6 and GH48), 11 β-glucosidases (three GH1 and eight GH3), 10 endo-1,4-β-xylanases (eight GH10, one GH11, and one GH43), two β-xylosidases (two GH39 and one GH43:1), four α-l-arabinofuranosidases (two GH43 and two GH51), two endo-1,5-α-arabinosidases (GH43), and an α-glucuronidase (GH67). These results are consistent with the cellulolytic and xylanolytic activities of this bacterial isolate.

The genome information will be useful for studies of microbial enzymes for industrial application in lignocellulosic biomass utilization.

### Accession number(s).

This whole-genome shotgun project has been deposited at NCBI SRA database under the accession no. LNTD00000000. The version described in this paper is version LNTD01000000.
